# Retrospective analysis of surgical outcomes for atlantoaxial subluxation

**DOI:** 10.1186/s13018-019-1112-2

**Published:** 2019-03-07

**Authors:** Tsuyoshi Yamada, Toshitaka Yoshii, Yu Matsukura, Takuya Oyaizu, Masato Yuasa, Takashi Hirai, Kyohei Sakaki, Hiroyuki Inose, Ichiro Torigoe, Kenichiro Sakai, Atsushi Okawa, Yoshiyasu Arai

**Affiliations:** 1 Department of Orthopaedic Surgery, Graduate School, Tokyo Medical and Dental University, 1-5-45 Yushima, Bunkyo-ku, Tokyo, 113-8510 Japan; 2 Department of Orthopaedic Surgery, Saiseikai Kawaguchi General Hospital, 5-11-5 Nishikawaguchi, Kawaguchi City, Saitama 332-8558 Japan

**Keywords:** Atlantoaxial subluxation, Laminectomy, Fixation, Atlantodental interval, Spine surgery

## Abstract

**Background:**

Atlantoaxial subluxation (AAS) is characterized by excessive movement at the junction between the atlas (C1) and axis (C2) as a result of either a bony or ligamentous abnormality. Surgical intervention is a therapeutic choice for AAS. In addition to C1 laminectomy (LAM), surgical fixation for subluxation or instability is performed by various techniques. While surgical treatment options for AAS have increased, the outcomes of different surgical techniques remain unclear.

**Methods:**

The authors conducted a retrospective analysis of the outcomes of 30 consecutive spinal surgeries performed for AAS patients, C1 LAM in 11 cases and C1/2 fixation in 19 cases. We investigated the correlation between the clinical outcomes and the surgical methods. We also examined the factors related to poor outcomes (the recovery rate of the Japanese Orthopedic Association score for cervical myelopathy < 40%) following AAS surgeries**.**

**Results:**

From a surgical method perspective, the patients in the C1 LAM group were older than those in the C1/2 fixation group (74.6 years vs 68.0 years), and the average recovery rate from the preoperative status was as follows: the C1 LAM group, 39.4%; the C1/2 fixation group, 49.8%. The C-JOA score was significantly improved after surgery in the C1/2 fixation group (from 9.8 to 13.1 points). The fixation technique seemed to successfully reduce C1/2 displacement. Each group exhibited a slight increase in the C1/2 angle and a decrease in the C2–7 angles after the operation. A higher preoperative atlantodental interval (ADI) was associated with good outcomes after the C1/2 fixation. The postoperative ADI was significantly reduced from 8.6 mm to 3.8 mm in the good outcome group after fixation. Patients with higher C1/2 angle showed good outcomes after C1 LAM. Despite the good neurological improvement, the C1/2 fixation method showed higher complication rates compared with C1 LAM method.

**Conclusions:**

The results of this study showed that the C1/2 fixation technique exhibited effectiveness in terms of neurological recovery. However, there was a high complication rate in surgeries for AAS, especially in the C1/2 fixation. C1 LAM would be considered for high-risk AAS cases such as elderly patients with multiple comorbidities.

## Introduction

Atlantoaxial subluxation (AAS) is characterized by excessive movement at the junction between the atlas (C1) and axis (C2) as a result of either a bony or ligamentous abnormality. Rheumatoid arthritis (RA) has been associated with a high incidence of progressive destruction of multiple joints, and it is one of the major causes of AAS. In addition to these inflammatory processes, congenital, traumatic, and neoplastic processes can result in subluxation and instability. These anatomical deformities may cause spinal cord or brain stem compression with resultant irreversible neurological deficits, such as cervical myelopathy, paresis, respiratory dysfunction, and even consequent death [1]. Further, because the vertebral arteries (VAs) are located near the atlantoaxial joint, thromboembolic stroke related to positional and transient VA occlusions can also occur [2–4]. Early diagnosis and treatment should be priorities in AAS patients.

Surgical intervention is a therapeutic choice for AAS. In addition to C1 laminectomy (LAM), surgical fixation for subluxation or instability is performed by various techniques. Recently, in the posterior fusion procedure for AAS, fixation using either a lateral mass [5, 6], a pedicle [7], a lamina of C2 [8], or transarticular [9] screws has been widely used, although the insertion of these screws carries a risk of injuring either the adjacent VA or spinal cord due to anatomical deformities or variations in C1/C2 or the VAs. While surgical treatment options for AAS have increased, the outcomes of different surgical techniques remain unclear, and few studies have compared clinical outcomes and perioperative complications among the surgical techniques used in AAS patients. In this study, we evaluated the clinical outcomes, including perioperative complications and radiographic findings, of spinal surgery in AAS patients. We further compared the clinical outcomes among surgical methods.

## Materials and methods

The current retrospective study was approved by our institutional review board. The authors performed a retrospective analysis of the outcomes of 30 consecutive spinal surgeries performed in AAS patients from February 2012 to August 2017. There were 19 males and 11 females, with an average age of 70.3 years (49–84 years) (Table 1).

**Table 1:** Demographics

Case, no.	30
RA, no.	7
OA, no.	20
Trauma, no.	3
Age, average, years	70.3 ± 8.7
Sex, female/male, no.	11/19
Ranawat value, average, mm	
Preoperative	14.0 ± 3.3
Postoperative	13.6 ± 2.7
CAA, average, degrees	
Preoperative	155.6 ± 7.4
Postoperative	158.4 ± 6.7
C1/2 angle, average, degrees	
Preoperative	19.9 ± 10.0
Postoperative	22.1 ± 9.4
ADI flexion/extension, average, mm	
Preoperative	8.1 ± 2.7/4.4 ± 2.4
Postoperative	5.5 ± 3.2/4.4 ± 2.6
ADI neutral, average, mm	
Preoperative	7.1 ± 2.8
Postoperative	4.7 ± 2.8^*^
C2–7 angles, average, degrees	
Preoperative	9.6 ± 13.6
Postoperative	3.5 ± 13.4
Cervical SVA, average, mm	
Preoperative	22.2 ± 19.6
Postoperative	26.0 ± 20.7
Operative time, average, min	163.5 ± 64.9
Blood loss, average, mL	136.5 ± 304.0
C-JOA score, average, points	
Preoperative	9.4 ± 3.9
Postoperative	12.4 ± 3.6^*^
Recovery rate of C-JOA score, average, %	46.0 ± 35.3
Complications, no.	9 (30.0%)
Revision, no.	6 (20.0%)

*RA* indicates rheumatoid arthritis, *OA* osteoarthritis, *CAA* clivoaxial angle, *ADI* atlantodental interval, *SVA* sagittal vertical axis, *C-JOA* Japanese Orthopedic Association for Cervical myelopathy

^*^p < 0.05 when compared to the preoperative status

AAS was diagnosed by a lateral cervical radiograph showing an anterior atlantodental interval (ADI) on a flexion radiograph of 5 mm or more [10, 11]. In patients with cervical myelopathy and/or severe neck pain due to C1/2 instability, surgery was usually indicated. The surgical methods were determined by the surgeons based on the patient’s age, general condition, comorbidities, instability, and difficulty of screw insertion. In principle, C1/2 fixation technique was applied for AAS with severe instability. In case with severe spinal cord compression in the neutral position, we added C1 LAM to the C1/2 fixation. C1 LAM alone was generally selected for aged AAS patients with comorbidities, especially for cases with small ADI and/or mild instability at C1/2.

**Fig. 1:**
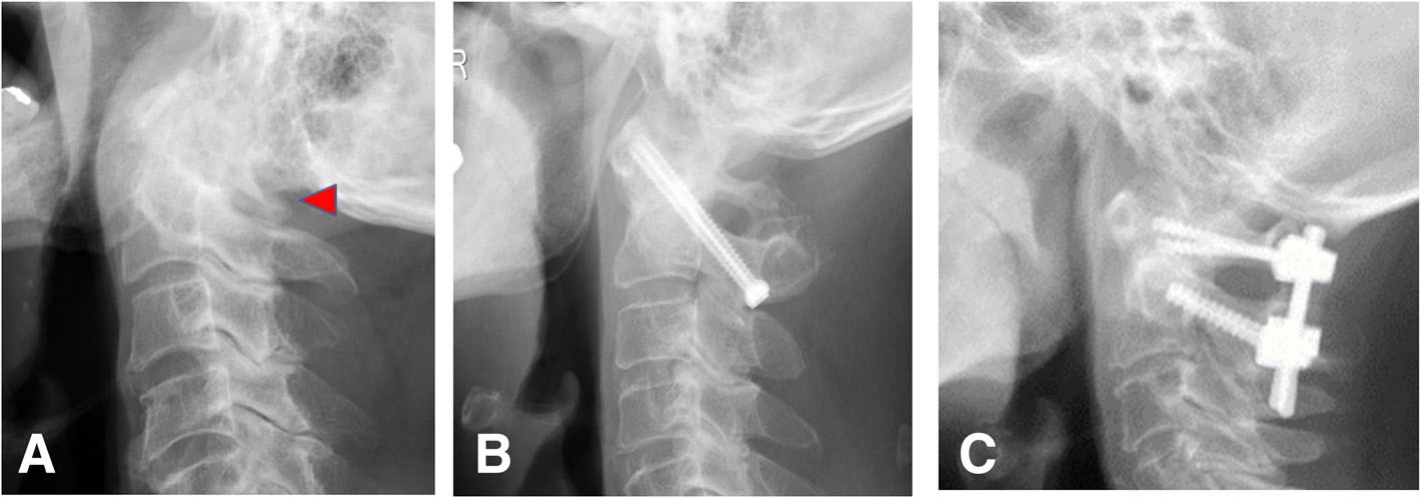
Representative lateral view of cervical spine X-rays post-operation. **a** C1 laminotomy. Posterior arch of C1 is decompressed (arrow head). **b** Transarticular screw fixation (Magerl and Brooks technique). **c** C1/2 fixation using C1 lateral mass and C2 pedicle screw

The preoperative comorbidities recorded in this study included diabetes mellitus in two patients, hypertension in two patients, pituitary disease in one patient, Down’s syndrome in one patient, arteriosclerosis obliterans (ASO) in one patient, atrial fibrillation (Af) in three patients, rheumatoid arthritis (RA) in seven patients, cerebral palsy (CP) in two patients, and polymyalgia rheumatic (PMR) in one patient. The causes of AAS in this study were as follows: trauma, 3 patients (10.0%); rheumatoid arthritis (RA), 7 patients (23.3%); and osteoarthritis (OA), 20 patients (66.7%, including one adjacent segment disorder and 2 CP patients). Five cases with retro-odontoid pseudotumor were found in OA patients. We performed C1 laminectomy (C1LAM) in 11 patients (Fig. 1a), C1/2 fixation using bilateral transarticular screws [Magerl (and Brooks) technique] [9, 12] in 5 patients (Fig. 1b), and C1/2 fixation using a C1 lateral mass and C2 pedicle/laminar screws [Tan (and Wright) technique] [5, 8] in 14 patients (Fig. 1c). Seven cases received C1 LAM in addition to C1/2 fixation (19 patients).

In general, the patients got out of bed on postoperative day 2 and started rehabilitation therapies. For external spine stabilization, in the patients treated with cervical fusion, a hard cervical collar was applied for 3 months. In patients who received laminectomy, a soft neck collar was used until discharge. In all these AAS patients, the cause of AAS, radiological findings, operative procedure, operation time, blood loss, clinical outcomes, and perioperative complications were reviewed. According to the radiographs of these patients, we retrospectively investigated the ADI at the flexion/extension position, Ranawat value, clivoaxial angle (CAA), C1/2 angle, C2–7 angles at the neutral position, and cervical sagittal vertical axis (SVA) in lateral cervical radiographs before surgery and at the last follow-up. The clinical outcomes were assessed by means of the scoring system proposed by the Japanese Orthopedic Association (JOA): the recovery rate in cervical cases: [(postoperative score–preoperative score)/(17-preoperative score)] ×  100% [13]. The surgical outcomes were classified into two groups: good outcomes included patients with a recovery rate of the C-JOA score higher than 40%; poor outcomes included patients with a recovery rate less than 40%.

We retrospectively compared the C1 LAM method and the C1/2 fixation method in terms of multiple clinical parameters (Table 2). We also examined the factors related to poor outcomes following AAS surgeries (Table 3). Statistical analysis was performed using Mann-Whitney *U* tests for non-normally distributed variables and chi-squared tests for categorical variables. All data are expressed as the mean ± standard deviation (SD). A *p* value less than 0.05 was considered to indicate a statistically significant difference.

**Table 2:** Comparison between the cases treated by C1 laminectomy and the cases treated by the C1/2 fixation

Surgery type	C1 laminectomy	C1/2 fixation
Case, no.	11	19
RA, no.	1 (9.1%)	6 (31.6%)
OA, no.	8 (72.7%)	12 (63.2%)
Trauma, no.	2 (18.2%)	1 (5.3%)
Age, average, years	74.6 ± 6.8	68.0 ± 9.0^†^
Sex, female/male, no.	2/9	9/10
Operative time, average, min	123.5 ± 54.9	186.6 ± 59.8^†^
Blood loss, average, mL	53.2 ± 69.2	184.7 ± 373.6
Ranawat value, average, mm		
Preoperative	13.8 ± 3.2	14.2 ± 3.5
Postoperative	13.1 ± 2.9	13.9 ± 2.6
CAA, average, degrees		
Preoperative	154.3 ± 7.5	156.4 ± 7.4
Postoperative	155.3 ± 5.9	160.3 ± 6.5^†^
C1/2 angle, average, degrees		
Preoperative	24.1 ± 10.8	17.5 ± 8.9
Postoperative	25.4 ± 10.5	20.2 ± 8.3
ADI flexion/extension, average, mm		
Preoperative	7.2 ± 2.4/5.0 ± 2.4	8.6 ± 2.8/4.0 ± 2.4
Postoperative	8.2 ± 2.9/5.4 ± 2.9	3.9 ± 2.2/3.9 ± 2.2
ADI neutral, average, mm		
Preoperative	6.1 ± 2.5	7.6 ± 2.9
Postoperative	6.1 ± 3.2	3.9 ± 2.2^*♰^
C2–7 angles, average, degrees		
Preoperative	10.5 ± 13.0	9.1 ± 14.2
Postoperative	2.8 ± 13.9	3.8 ± 13.4
Cervical SVA, average, mm		
Preoperative	40.6 ± 13.8	12.3 ± 14.4^†^
Postoperative	41.9 ± 20.7	17.5 ± 15.4^†^
C-JOA score, average, points		
Preoperative	8.5 ± 4.1	9.8 ± 3.8
Postoperative	11.1 ± 4.2	13.1 ± 3.1^*^
Recovery rate of C-JOA score, average, %	39.4 ± 30.5	49.8 ± 38.0
Complications, no.	Hematoma = 1 AAF = 1 (18.2%)	Septic emboli = 1 SSI = 2 DSI = 1 VS = 1 AAF = 2 (36.8%)
Revision, no.	2 (18.2%)	4 (21.1%)

*RA* indicates rheumatoid arthritis, *OA* osteoarthritis, *CAA* clivoaxial angle, *ADI* atlantodental interval, *SVA* sagittal vertical axis, *C-JOA* Japanese Orthopedic Association for Cervical myelopathy, *SSI* surgical site infection, *DSI* donor site infection, *VS* vertical subluxation, *AAF* Anterior arch fracture

^*^p < 0.05 when compared to the preoperative status

^†^*p* < 0.05 when compared to the C1 laminectomy group

**Table 3:** Comparison between the cases with poor clinical outcomes and the cases with good outcomes

	C1 laminectomy		C1/2 fixation	
Clinical outcome	Poor	Good	Poor	Good
Case, no.	6	5	7	12
RA, no.	1 (16.7%)	0 (0%)	3 (42.9%)	3 (25.0%)
OA, no.	4 (66.7%)	4 (80.0%)	4 (57.1%)	8 (66.7%)
Trauma, no.	1 (16.7%)	1 (20.0%)	0 (0%)	1 (8.3%)
Age, average, years	74.2 ± 7.9	74.8 ± 6.2	69.1 ± 10.5	67.3 ± 8.3
Sex, female/male, no.	1/5	1/4	3/4	6/6
Operative time, average, min	121.3 ± 62.7	126.2 ± 51.0	159.6 ± 47.4	202.4 ± 62.3
Blood loss, average, mL	61.0 ± 89.0	43.8 ± 43.1	149.4 ± 244.0	205.3 ± 441.1
Ranawat value, average, mm				
Preoperative	13.7 ± 3.0	13.8 ± 3.7	14.6 ± 4.6	14.0 ± 2.3
Postoperative	12.9 ± 3.2	13.3 ± 2.9	14.2 ± 3.2	13.7 ± 2.3
CAA, average, degrees				
Preoperative	157.2 ± 7.9	150.8 ± 5.8	154.3 ± 7.5	154.3 ± 8.2
Postoperative	155.3 ± 6.2	155.2 ± 6.2	155.3 ± 5.9	158.1 ± 7.2
C1/2 angle, average, degrees				
Preoperative	21.7 ± 6.4	27.0 ± 14.8	21.0 ± 3.4	15.5 ± 10.6
Postoperative	23.0 ± 8.6	28.3 ± 12.7	21.0 ± 9.4	19.7 ± 8.1
ADI flex/ext., average, mm				
Preoperative	7.8 ± 2.8/5.3 ± 2.9	6.5 ± 1.9/4.5 ± 1.9	7.7 ± 2.2/3.4 ± 1.7	9.1 ± 3.1/4.3 ± 2.7
Postoperative	8.3 ± 2.6/5.7 ± 3.1	8.0 ± 3.5/5.0 ± 2.9	3.9 ± 1.8/3.9 ± 1.3	3.9 ± 2.5/3.9 ± 2.5
ADI neutral, average, mm				
Preoperative	6.4 ± 3.1	6.1 ± 1.9	5.9 ± 1.3	8.6 ± 3.2^†^
Postoperative	6.3 ± 3.2	5.8 ± 3.6	3.9 ± 1.3^*^	3.8 ± 2.5^*^
ΔADI	0.2 ± 1.0	0.3 ± 1.9	2.0 ± 1.3	4.7 ± 3.5^†^
C2–7 angles, average, degrees				
Preoperative	12.2 ± 17.7	8.6 ± 4.5	4.9 ± 12.5	11.6 ± 15.1
Postoperative	5.8 ± 17.7	− 0.8 ± 7.8^*^	4.9 ± 8.7	3.1 ± 15.8
Cervical SVA, average, mm				
Preoperative	37.8 ± 15.8	43.5 ± 13.2	14.9 ± 12.6	10.6 ± 15.9
Postoperative	43.8 ± 21.5	40.0 ± 22.9	18.6 ± 18.2	16.8 ± 14.3
C-JOA score, average, points				
Preoperative	6.9 ± 3.5	10.5 ± 4.1	9.7 ± 3.6	9.9 ± 4.1
Postoperative	8.5 ± 3.2	14.3 ± 2.8^†^	10.6 ± 3.2	14.5 ± 2.0^*♰^
Recovery rate of C-JOA score, average, %	16.1 ± 6.8	67.4 ± 21.8^†^	11.7 ± 28.3	72.0 ± 21.7^†^
Complications, no.	AAF = 1 (16.7%)	Hematoma = 1 (20.0%)	Septic emboli = 1 SSI = 1 VS = 1 (42.9%)	SSI = 1 DSI = 1 AAF = 2 (33.3%)
Revision, no.	1 (16.7%)	1 (20.0%)	2 (28.6%)	3 (25.0%)

*RA* indicates rheumatoid arthritis, *OA* osteoarthritis, *CAA* clivoaxial angle, *ADI* atlantodental interval, *SVA* sagittal vertical axis, *C-JOA* Japanese Orthopedic Association for Cervical myelopathy, *SSI* surgical site infection, *DSI* donor site infection, *VS* vertical subluxation, *AAF* Anterior arch fracture

^*^p < 0.05 when compared to the preoperative status

^†^*p* < 0.05 when compared to the poor outcome group

## Results

The patients in this study tolerated the surgical procedure well and were followed up for an average of 3.4 years (1–6.5 years). The average ADI at the flexion/neutral/extension position was 8.1 mm/7.1 mm/4.4 mm preoperatively and 5.5 mm/4.7 mm/4.4 mm at the final follow-up. The C1/2 angle, C2–7 angles, and cervical SVA were 19.9°/9.6°/22.2 mm preoperatively and 22.1°/3.5°/26.0 mm at the last follow-up. The ADI at the neutral position of the neck was significantly improved (*p* = 0.015). Of note, there was a substantial difference between the preoperative and postoperative C2–7 angles (Table 1). Regardless of C1/2 fixation, 21 of 30 patients (70.0%) had a decreased C2–7 angles.

From a surgical method perspective, the age of the patients undergoing C1 LAM was higher than those undergoing C1/2 fixation (74.6 years vs. 68.0 years; *p* = 0.0469). The average recovery rate from the preoperative status was as follows: the C1 LAM group, 39.4%; the C1/2 fixation group, 49.8% (the Magerl method 41.1%; the Tan method 51.4%). The C-JOA score was significantly improved after surgery in the C1/2 fixation group (from 9.8 to 13.1 points; *p* = 0.006), although these fixation techniques needed a relatively longer operative time (*p* = 0.0078) and had a higher estimated blood loss than those of the C1 LAM group. The fixation technique seemed to successfully reduce C1/2 displacement and maintain a C1/2 angle of approximately 20°. The ADI at the neutral position of the neck was significantly shortened (*p* < 0.0001) and well stabilized in the C1/2 fixation group. However, the C1 LAM group did not show a reduction in C1/2 displacement, leading to an average ADI of 6.1 mm at the neutral position during the follow-up period. While the Ranawat value and CAA were not different before and after surgery, each group exhibited a slight increase in the C1/2 angle and a decrease in the C2–7 angles after the operation. Regarding cervical sagittal balance, the cervical SVA in the C1 LAM group was greater than that in the C1/2 fixation group before and after the operation (*p* = 0.0002 and *p* = 0.0042, respectively) (Table 2).

Perioperative surgical complications occurred in 9 patients (30.0%). In terms of postoperative complications, neurological deterioration due to hematoma occurred in 1 patient (9.1%) and anterior arch fracture of C1 (AAF) occurred in 1 CP patient (9.1%) in the C1 LAM group, while 7 complications (36.8%) were observed in the fixation group: severe pneumonia leading to septic emboli in 1 patient, remarkable vertical subluxation (VS) in 1 patient, AAF in 2 patients (1 trauma and 1 non-trauma), deep surgical site infection (SSI) in 2 patients, and donor site infection (DSI) in 1 patient. Of note, septic emboli observed in the fixation group induced acute cerebral infarctions, leading to neurological deterioration. Revision surgeries were performed in 6 of the 30 patients (20.0%). One hematoma (3.3%), 2 SSI (6.7%), and 1 DSI (3.3%) cases required open irrigation with aggressive surgical debridement. An additional C1/2 fixation (3.3%) was performed due to severe neck pain after AAF in the C1 LAM group while an additional corrective occipital-cervical spine fusion (3.3%) was applied due to progressive VS in the fixation group. The complication and revision rates in the C1/2 fixation group were higher than those in the C1 LAM group, although there were no significant differences between the two groups (Table 2).

According to the univariate analyses of surgical outcomes, a higher preoperative ADI and a good reduction of ADI were associated with good outcomes after C1/2 fixation (*p* = 0.0368 and *p* = 0.0253). While the postoperative ADI was not significantly different from the preoperative value in the poor outcome group, the postoperative ADI was significantly reduced from 8.6 mm to 3.8 mm in the good outcome group after the fixation (*p* = 0.0006). In patients who received C1 LAM, we found no significant differences in ADI and C1/2 translation between the good outcome group and the poor outcome group. However, preoperative C1/2 angle was smaller in the poor outcome group after C1 LAM. Other clinical parameters, radiological parameters, and causes were not statistically associated with poor outcomes regardless of the surgical procedure (Table 3).

## Discussion

In this study, we retrospectively investigated the multiple clinical outcomes from each surgical method in the C1 LAM group and the C1/2 fixation group. In the C1/2 fixation group, the ADI at the neutral position of the neck was significantly shortened with good stability compared with the C1 LAM group. Further, the C-JOA score was significantly improved after C1/2 fixation, in which the recovery rate of the C-JOA score reached 49.8%. In the C1 LAM group, neurological improvement was slightly less (39.4%) than that in the C1/2 fixation group. However, even in the C1 LAM group, the neurological scores were improved after surgery in all the patients and no patients showed neurological deterioration. Furthermore, as previously reported [14], C1 LAM without fixation did not induce C1/2 instability or imbalance of the cervical spine; increases in the ADI or SVA were not observed during the follow-up period. According to this result, C1/2 fixation is generally considered first for treatment of patients with AAS, but C1 LAM may be an option for high-risk AAS patients.

One of the adverse events following C1/2 fixation surgery is subaxial kyphotic changes. It has been reported that 33–48% of all patients who undergo atlantoaxial arthrodesis develop postoperative kyphosis or swan-neck deformity of the lower cervical spine. Several authors suggested that this postoperative kyphotic change is attributable to C1/2 fixation in a hyperextended position [15–19]. In our patients who underwent C1/2 fixation, we planned the surgeries with a fixation angle of approximately 20°, as recommended in the previous studies [15, 18, 20], and successfully achieved the optimal postoperative C1/2 angle (20° ± 5°). However, most of the patients in this study (70.0%) developed postoperative loss of lower cervical spine lordosis during the follow-up period. Even in the group without fixation (the C1 LAM group), subaxial kyphotic changes occurred after surgery. The invasion to upper cervical lesions itself may contribute to this kyphotic alignment change because the semispinalis cervical muscle needs to be partially removed during surgery. In AAS surgery, great care should be taken regarding the C1/2 angle and the invasion of the muscles attached to C2. In addition, postoperative therapy to prevent kyphotic changes is considered important.

In patients who received C1/2 fixation, higher preoperative ADI and good reduction of ADI were related to good surgical outcomes in high-ADI patients, anterior subluxation of C1, and instability at C1/2 are considered the main factors for neurological problems. Thus, a good reduction of the ADI is directly associated with decompression of the spinal cord and neurological recovery. However, in patients with a small ADI, other pathogens, such as periodontoid synovitis, may also cause the neurological impairment and thus the neurological recovery may not be as good as that in patients with a high preoperative ADI. In patients who received C1 LAM alone, we found no differences in ADI and C1/2 translation between the good outcome and poor outcome group probably because patients with relatively small ADI and mild instability were likely treated with C1 LAM in this study. However, preoperative C1/2 angle was smaller in the poor outcome group. The poor outcome after C1 LAM in cases with small C1/2 angle may be attributed to the steeper angle between a dens tip and a spinal cord [18], leading to the disadvantage to the neurological improvements. From these results, C1/2 fixation with reduction is generally recommended for patients with high preoperative ADI. For patients with small ADI, C1 LAM may provide favorable results if the patients have large C1/2 angle.

Spinal surgery in AAS patients has been considered challenging because most patients are elderly and have multiple medical problems. In the present study, severe complications such as hematoma, AAF, VS, cerebral infarctions, and infections after spinal surgeries occurred. The reoperation rate reached 20.0% in the AAS patients. In particular, the complication and revision rates in the C1/2 fixation group were higher than those in the C1 LAM group (complication rate 18.2% vs 36.8%, and revision rate 18.2% vs 21.1%, respectively). A high incidence of surgical site infections is also an issue in spinal operations for AAS patients. The AAS patients in this study included a large number of patients who had a high risk of developing SSI, including patients with diabetes mellitus and collagen diseases such as RA and patients taking other immunosuppressants. In addition, risk for skin contamination is high in the upper cervical region [21]. In this study, the rate of SSI was indeed high (6.7%). These results indicate that patients with AAS need a more careful risk assessment and risk management, especially those who undergo fixation surgery.

This study has several limitations, including the following: (1) patient baseline assessments were not controlled; (2) the number of patients was small; and (3) this was a retrospective investigation. These limitations could be partially ascribed to the challenges of AAS-related surgery. Because this study lacks a powerful statistical analysis, prospective data collection is needed in the future to clarify more precise clinical outcomes in AAS patients.

Despite these limitations, our results demonstrated important information regarding the surgical treatment of AAS. In our case series of surgically treated AAS, the C1/2 fixation technique exhibited effectiveness in terms of neurological recovery. For patients with high preoperative ADI, C1/2 fixation with reduction is generally recommended. However, patients with AAS, who undergo fixation surgery, need a careful risk assessment and risk management because of the high complication rate. In cases with small ADI and mild instability, C1 LAM may provide favorable results if the patients have large C1/2 angle. As perioperative complication in C1 LAM is less than in C1/2 fixation, C1 LAM would be considered for high-risk AAS cases such as elderly patients with multiple comorbidities.

## Conclusion

In our case series of surgically treated AAS, the C1/2 fixation technique exhibited effectiveness in terms of neurological recovery. For patients with high preoperative ADI, C1/2 fixation with reduction is generally recommended. In cases with small ADI and mild instability, C1 LAM may provide favorable results if the patients have large C1/2 angle. Because of less complication rate, C1 LAM would be considered for high-risk AAS cases such as elderly patients with multiple comorbidities.

## Abbreviations

AAF: Anterior arch fracture
AAS: Atlantoaxial subluxation
ADI: Atlantodental interval
Af: Atrial fibrillation
ASO: Arteriosclerosis obliterans
CAA: Clivoaxial angle
CP: Cerebral palsy
DSI: Donor site infection
JOA: Japanese Orthopedic Association
LAM: Laminectomy
OA: Osteoarthritis
PMR: Polymyalgia rheumatic
RA: Rheumatoid arthritis
SD: Standard deviation
SSI: Surgical site infection
SVA: Sagittal vertical axis
VA: Vertebral artery
VS: Vertical subluxation
